# Quantifying human genome parameters in aging

**DOI:** 10.18699/VJGB-23-60

**Published:** 2023-09

**Authors:** V.P. Volobaev, S.S. Kunizheva, L.I. Uralsky, D.A. Kupriyanova, E.I. Rogaev

**Affiliations:** Sirius University of Science and Technology, Scientific Center for Genetics and Life Sciences, Sochi, Russia; Sirius University of Science and Technology, Scientific Center for Genetics and Life Sciences, Sochi, Russia Vavilov Institute of General Genetics, Russian Academy of Sciences, Department of Genomics and Human Genetics, Moscow, Russia Lomonosov Moscow State University, Center for Genetics and Genetic Technologies, Faculty of Biology, Moscow, Russia; Sirius University of Science and Technology, Scientific Center for Genetics and Life Sciences, Sochi, Russia Vavilov Institute of General Genetics, Russian Academy of Sciences, Department of Genomics and Human Genetics, Moscow, Russia; Sirius University of Science and Technology, Scientific Center for Genetics and Life Sciences, Sochi, Russia; Sirius University of Science and Technology, Scientific Center for Genetics and Life Sciences, Sochi, Russia Vavilov Institute of General Genetics, Russian Academy of Sciences, Department of Genomics and Human Genetics, Moscow, Russia Lomonosov Moscow State University, Center for Genetics and Genetic Technologies, Faculty of Biology, Moscow, Russia University of Massachusetts Chan Medical School, Department of Psychiatry, Shrewsbury, USA

**Keywords:** genome quantification, aging, longevity, neurodegenerative disorders, mtDNA, telomere length, somatic mutations., количественные параметры генома, старение, долголетие, нейродегенеративные заболевания;, mtDNA, длина теломер, соматические мутации

## Abstract

Healthy human longevity is a global goal of the world health system. Determining the causes and processes influencing human longevity is the primary fundamental goal facing the scientific community. Currently, the main efforts of the scientific community are aimed at identifying the qualitative characteristics of the genome that determine the trait. At the same time, when evaluating qualitative characteristics, there are many challenges that make it difficult to establish associations. Quantitative traits are burdened with such problems to a lesser extent, but they are largely overlooked in current genomic studies of aging and longevity. Although there is a wide repertoire of quantitative trait analyses based on genomic data, most opportunities are ignored by authors, which, along with the inaccessibility of published data, leads to the loss of this important information. This review focuses on describing quantitative traits important for understanding aging and necessary for analysis in further genomic studies, and recommends the inclusion of the described traits in the analysis. The review considers the relationship between quantitative characteristics of the mitochondrial genome and aging, longevity, and age-related neurodegenerative diseases, such as the frequency of extensive mitochondrial DNA (mtDNA) deletions, mtDNA half-life, the frequency of A>G replacements in the mtDNA heavy chain, the number of mtDNA copies; special attention is paid to the mtDNA methylation sign. A separate section of this review is devoted to the correlation of telomere length parameters with age, as well as the association of telomere length with the amount of mitochondrial DNA. In addition, we consider such a quantitative feature as the rate of accumulation of somatic mutations with aging in relation to the lifespan of living organisms. In general, it may be noted that there are quite serious reasons to suppose that various quantitative characteristics of the genome may be directly or indirectly associated with certain aspects of aging and longevity. At the same time, the available data are clearly insufficient for definitive conclusions and the determination of causal relationships.

## Abstract (ru)

Здоровое долголетие человека – глобальная цель мировой системы здравоохранения. В то же время неуклонное старение населения стало серьезным вызовом для систем здравоохранения многих стран мира, в том числе из-за возросшего риска развития многих тяжелых нейродегенеративных заболеваний, включая болезнь Альцгеймера (БА) и болезнь Паркинсона (БП). Определение причин и процессов, влияющих на старение и продолжительность жизни человека, а также выявление механизмов развития возрастных патологий – первостепенная фундаментальная задача, стоящая перед научным сообществом. В настоящее время основные усилия направлены на идентификацию качественных характеристик генома, детерминирующих признак. Вместе с тем при их оценке существует множество проблем, затрудняющих установление ассоциаций. Количественные признаки обременены таковыми проблемами в меньшем объеме, но в большинстве случаев упускаются при проведении современных геномных исследований, посвященных вопросам старения и долголетия. Несмотря на наличие широкого круга возможностей проведения анализа геномных данных по количественным признакам, большинство возможностей не используется, что наряду с недоступностью опубликованных данных ведет к потере этой важной информации. Настоящий обзор посвящен описанию количественных признаков, важных для понимания процесса старения и необходимых для анализа в дальнейших геномных исследованиях, и является рекомендацией для включения описанных признаков в анализ. Рассматривается взаимосвязь количественных характеристик ядерного и митохондриального генома со старением, долголетием и возрастными нейродегенеративными заболеваниями, таких как частота обширных делеций митохондриальной ДНК (mtDNA), время полураспада mtDNA, частота замен A>G в тяжелой цепи mtDNA, количество копий mtDNA, длина теломер, частота соматических мутаций. В целом можно отметить, что есть достаточно серьезные причины полагать, что различные количественные характеристики генома могут быть прямо или косвенно ассоциированы с теми или иными аспектами старения и продолжительности жизни. Но имеющихся данных недостаточно для окончательных выводов и выявления причинно-следственных связей.

## Introduction

Human longevity is a complex trait that is influenced by environmental factors, lifestyle, random events, and individual genetic traits. Studies have shown that genetics plays a significant role in longevity, with individuals from families of longlivers having a higher chance of living longer (van den Berg, 2020). However, identifying specific genetic determinants associated with longevity has been challenging. Currently, only two genes, APOE and FOXO3A, have been shown to be important for human longevity across different samples and research groups (Deelen et al., 2019). Other results have been inconsistent, possibly due to population differences and the effect of multiple comparisons.

Despite the difficulty in identifying specific genetic determinants, maintaining body health is crucial for longevity. Aging of the brain and the development of cerebrovascular and neurodegenerative diseases are major causes of disability and death in older adults (Debette et al., 2019). However, the genetic basis of age-related brain diseases is complex and inconsistent. In contrast to qualitative characteristics of the genome, quantitative traits such as telomere length, the number of mitochondrial DNA copies, the frequency of heterozygous variants of mitochondrial DNA, and the frequency of somatic mutations are less affected by population and statistical factors. Despite their importance, little attention has been paid to these quantitative traits in the study of the genetic basis of longevity

This review aims to analyze existing information on quantitative genetic traits affecting aging and human longevity.

## Quantification of changes in mtDNA structure due to aging

In recent years, there has been much interest in the role of mtDNA as a determinant of aging, lifespan processes, and agerelated diseases. Mitochondrial dysfunction is considered one of the key aging biomarkers (Miva et al., 2022), and changes in quantitative and qualitative characteristics of mtDNA are directly associated with longevity. Given the spatial proximity of mtDNA to the electron transport chain, it is exposed to the damaging effects of free radicals, which, along with a limited ability to repair, due to the fact that mtDNA is not protected by histones and is in a single-stranded form for a considerable part of its replication time, determines the vulnerability of mtDNA structure to damage and degradation. All of these factors lead to a higher rate of chemical modifications and mutation accumulation in mtDNA compared to the cell nucleus DNA.

Damage and deletion of mtDNA sites can lead to mitochondrial dysfunction due to an increased proportion of molecules containing an extensive deletion (mtDNAdel), since mtDNA with an extensive deletion has a replicative advantage over wild-type mtDNA (Kowald, Kirkwood, 2018). The replicative advantage is probably determined by the smaller size of the replicating molecule, which leads to a higher replication rate (Diaz, 2002), and at the same time, a lower chance of damage to the molecule by active oxygen species. This results in less active mitophagy of mtDNAdelrich organelles compared to normal organelles (de Grey, 1997). Moreover, whereas in actively proliferating tissues, cells containing dysfunctional mitochondria are subject to elimination and replacement, tissues characterized by a high number of postmitotic cells accumulate a burden of such mutations, which probably leads to a decrease in the functional parameters of the tissue (Herbst et al., 2017).

The proportion of mtDNAdel in muscle tissue has been shown to increase approximately 19-fold, from 0.008 to 0.15 %, from 50 to 86 years (Herbst et al., 2021b). A similar phenomenon has been observed in nerve tissue (Nido et al., 2018). It has also been noted that significant accumulation of mtDNAdel is observed in patients with Parkinson’s disease in substantia nigra neurons (Bender et al., 2006; Grünewald et al., 2016) and the striatum (Ikebe et al., 1990). Moreover, there is an opinion that accumulation of mtDNAdel can trigger neuroprotective mechanisms (Perier et al., 2013).

The state of mtDNA heterogeneity in which several clones of mtDNA with different nucleotide sequences exist in mitochondria is called heteroplasmy. It is known that heteroplasmy can occur either de novo during ontogenesis or by maternal inheritance (Sallevelt et al., 2017). Heteroplasmic mutations also appear to be associated with macroinflammation (Just et al., 2015). For example, R. Zhang and colleagues noted that, on average, individuals over the age of 70 had 58.5 % more mtDNA heteroplasmic mutations than individuals under the age of 40 (Zhang et al., 2017). This fact becomes of great significance when we consider that there is substantial evidence linking mtDNA heteroplasmy with neurodegenerative diseases directly associated with longevity: Alzheimer’s disease (AD) (Tranah et al., 2012) and Parkinson’s disease (PD) (Hudson et al., 2013). At the same time, there are reports showing a positive role of heteroplasmy for longevity (Rose et al., 2010; Sondheimer et al., 2011), which is probably because mtDNA heteroplasmy is a reservoir of genetic variability that can introduce new functions and increase the ability of cells to cope with environmental and physiological stressors during life. It can be assumed that both of these phenomena take place and their importance for longevity is determined by the localization of somatic mtDNA mutation accumulation and by the fact that congenital heteroplasmy can have a positive effect to a greater extent, while acquired one has a greater chance to carry negative properties.

Another mitochondrial marker likely associated with longevity may be the frequency of accumulation of mitochondrial somatic mtDNA (mtSNV) A>G mutations in the mtDNA heavy chain. In a recent study (Mikhailova et al., 2022), the authors determined a positive correlation between the frequency of AH>GH (H – heavy chain) substitutions and the lifespan of different mammalian species: the more long-lived a species is, the higher the frequency of AH>GH substitutions is observed in it. At the same time, the authors suggest that the observed accumulation of GH nucleotides is a consequence of oxidative mutagenesis and aging processes rather than a cause.

The half-life of mtDNA also seems to be an important factor determining the rate of tissue dysfunction onset. It has been suggested that cell lifespan depends on mtDNA half-life (Poovathingal et al., 2012; Chan et al., 2013). In modeling the effect of half-life on cell survival time, it has been determined that a moderate increase in mtDNA half-life has a profound effect on increasing cell survival time, thereby reducing the replicative advantage of mtDNA with extensive deletions (Holt, Davies, 2021). Equally importantly, a decrease in mtDNA half-life significantly affects the process of mtSNV accumulation in tissues characterized by a high number of postmitotic cells. It has been shown that if the half-life is three months, pathogenic mtSNV acquired in a neuronal progenitor cell early in development and present in the postmitotic neuronal population at an average frequency of 1 %, by 70 years of human life, will be contained in most neurons with a frequency of ~14 % (Li et al., 2019). Accordingly, changing the half-life rate downward acts to inhibit mitochondrial heteroplasmy levels and vice versa.

In addition to mutational events, the mtDNA copy number (mtDNAcn) is an important quantitative trait. Changes in mtDNAcn are usually a reflection of the mitochondrial response to oxidative stress and are also associated with general dysfunction. Various studies have reported results showing a decrease in mtDNAcn as humans age (Herbst et al., 2017, 2021a). A decrease in mtDNA copies in whole blood has been found to occur with age, and a lower number of mtDNA copies is associated with poorer health (Lee et al., 2010; Mengel-From et al., 2014). High mtDNAcn levels are probably generally associated with better health outcomes in older individuals, including higher levels of cognitive function and lower mortality (Kim et al., 2013; Mengel-From et al., 2014). It has been noted that a decreased mtDNAcn score is strongly associated with the risk of age-related neurodegenerative diseases such as dementia, PD, AD, etc. (Yang et al., 2021).

It should be noted that both systemic trends toward a decrease in mtDNAcn in individuals with AD and a local decrease in mtDNAcn by 30–50 % in the frontal lobe of the large hemisphere cortex and hippocampus compared to healthy controls have been observed (Coskun et al., 2004; Rice et al., 2014). At the same time, there is a publication that describes an increase in mtDNAcn in patients of African descent with Parkinson’s disease (Müller-Nedebock et al., 2022).

In studies examining changes in mtDNAcn in the blood leukocytes of long-livers as a model of healthy aging, contradictory results have been obtained. Y.H. He et al. (2014) showed a significant increase in the amount of mtDNAcn in centenarians compared to elderly people (He et al., 2014), but van Leeuwen et al. did not observe such a pattern (van Leeuwen et al., 2014), which may be due to different methodological approaches. It should be noted that different tissues may show different age dynamics of mtDNAcn. For example, while an inverse correlation was observed in skeletal muscle samples, a positive correlation was observed in liver or substantia nigra samples (Dölle et al., 2016; Wachsmuth et al., 2016).

The mtDNAcn index seems to be related to the telomere length (TL) parameter (Qiu et al., 2015; Tyrka et al., 2015; Dolcini et al., 2020). It is assumed that this relationship is based on the negative correlation between mtDNAcn levels and levels of reactive oxygen species (ROS) and further negative effects of ROS on telomere length (Melicher et al., 2018).

## Telomere length as a cause or consequence of longevity

Telomere length is a well-known biomarker of aging (Sanders, Newman, 2013). Although the relationship between TL and cellular aging is undeniable in model cell cultures (Victorelli, Passos, 2017), the conclusions for multicellular organisms are not so unambiguous (Blackburn et al., 2015). It has been suggested that telomere shortening dynamics, rather than total telomere length, can serve as a quantitative biomarker of macroorganism lifespan (Vera et al., 2012). For example, in cross-sectional studies on five bird species, it was shown that short-lived bird species lose more telomere repeats with age than species with longer lifespans (Haussmann et al., 2003). A similar correlation has been observed in mammals, suggesting that long-lived animals have more effective mechanisms of protection against replicative aging, such as higher telomerase activity throughout life (Haussmann et al., 2007).

In humans, shorter telomere length is associated with higher mortality rates from various age-related pathologies, including some neurodegenerative diseases such as dementia (Levstek et al., 2021). However, reports on the role of telomere length in the risk of AD are ambiguous. Some studies noted that TL length is lower in people with AD than in control samples (Thomas et al., 2008; Forero et al., 2016), while P. Thomas et al. noted an inverse relationship in some tissues such as the hippocampus. Interestingly, longer telomeres have a negative effect on disease dynamics and severity (Movérare-Skrtic et al., 2012; Mahoney et al., 2019). Short TL is a good prognostic marker for determining the long-term risk of AD in APOE4- negative individuals (Hackenhaar et al., 2021). Moreover, TL is associated with cognitive function in both elderly and middle-aged individuals (Hägg et al., 2017; Gampawar, 2022).

It has been estimated that leukocyte telomeres in adults shorten at an average rate of 24.7 bp per year (Müezzinler et al., 2013). A number of different factors can influence TL and the rate of telomere depletion. For example, TL has been shown to be higher in older women compared to men (Benetos et al., 2001) and in African Americans compared to Caucasians (Hunt et al., 2008). First of all, it should be noted that in addition to the large number of studies that have noted a negative correlation of TL with age and the association of this parameter with mortality in the older age group, there are also studies in which these patterns were not confirmed (Sanders, Newman, 2013).

Initially, it was assumed that such discrepancies are associated with the peculiarities of specific studies, such as the methodology of sample formation, the presence of population stratification, the type of tissue studied, and the methods of studying the index. For example, in an extensive study of TL in various tissues, it was determined that in 21 types of tissue, there is a negative correlation of TL with age (the strongest correlations for whole blood and gastric tissue), while no correlation was observed for testes, ovaries, cerebellum, vagina, skeletal muscle, thyroid gland, and gastroesophageal junction tissue (Demanelis et al., 2020).

When studying long-livers as a model of healthy aging, it was hypothesized that TL primarily depends on the physiological state of the organism rather than age. It was shown that in “high-performing” long-livers (with a low number of diseases and high physical activity), TL was significantly higher than TL in “low-performing” long-livers (with a high number of diseases and low physical activity). Therefore, it has been suggested that it is probably not the telomere length factor that affects the ability to live to one hundred years, but the health condition associated with telomere length (Terry et al., 2008; Tedone et al., 2019). This theory is also supported by a study of TL in same-sex twins over the age of 70, which noted a clear association between blood white cell TL and physical health, including between twins (Bendix et al., 2011). Thus, the study of telomere dynamics in long-lived individuals is of particular importance because they may have developed mechanisms that actively postpone aging and provide effective protection against the negative effects of aging processes.

## Somatic mutations and their role in longevity

The current theory of aging suggests that the accumulation of DNA mutations in somatic cells (copy number variations, CNVs) with age leads to a decrease in cell function due to the inactivation or disruption of important genes (Kennedy et al., 2012). Indeed, it has been shown that the accumulation of somatic mutations occurs with age and at a differential rate for different tissues. For example, in human proximal bronchial basal cells, the rate of mutation accumulation is approximately 29 CNVs per cell per year (CNVs/pcpy) (Huang et al., 2022); in prefrontal cortex and hippocampal neurons, it is 16–21 CNVs/pcpy (Lodato et al., 2018; Miller et al., 2022); in subcutaneous preadipocytes, it is 18 CNVs/pcpy; in visceral adipose tissue preadipocytes, it is 27 CNVs/pcpy (Franco et al., 2019); in memory T cells, it is approximately 25 CNVs/pcpy; in naive B-lymphocytes, it is approximately 15 CNVs/pcpy; in hematopoietic stem cells and progenitor cells, it is approximately 16 CNVs/pcpy (Machado et al., 2022); and in spermatogonia, it is approximately 2 CNVs/pcpy (Milholland et al., 2017).

A vivid illustration of the significance of CNVs for lifespan is provided by studies of the rate of mutation accumulation in the crypts of the large intestine in mammals with different lifespans (Cagan et al., 2022). While the rate for humans is approximately 47 CNVs/pcpy, for giraffes at 25–35 years of life, it is approximately 99 CNVs/pcpy; for ferrets at 14 years of life, it is approximately 496 CNVs/pcpy; and for mice at 2 years of life, it is approximately 796 CNVs/pcpy. Thus, the dependence of lifespan and the rate of mutation accumulation is well established.

At the same time, it has been shown that the frequency of somatic mutations in humans in old age is much lower than that required for the loss of gene function in a significant number of cells, indicating an indirect relationship between the indices (Vijg, Dong, 2020). In a study of a large sample of Chinese centenarians compared to controls, it was observed that CNV levels were significantly higher in the sample of centenarians than in the control sample, indicating that the frequency of CNVs does not directly affect the probability of living beyond the population norm (Zhao et al., 2018). On the other hand, a study of centenarians from Italy obtained different data, observing that centenarians had significantly lower levels of CNVs than controls (Garagnani et al., 2021). Given the contradictory results obtained in these two studies, more research on this issue is needed.

## Conclusion

Thus, there are reasons to suggest that there is a significant association between aging dynamics, life expectancy, healthy aging, the risk of neurodegenerative diseases, and various quantitative genomic characteristics. At the same time, what is the cause and what is the effect in most cases is not determined, which, along with the sporadic nature of the available publications, highlights the need for additional research.

## Conflict of interest

The authors declare no conflict of interest.
